# Acidosis regulates immune progression in rheumatoid arthritis by promoting the expression of cytokines and co-stimulatory molecules in synovial fibroblasts

**DOI:** 10.1186/s10020-025-01181-x

**Published:** 2025-04-15

**Authors:** Xuewen Qian, Zhuoyan Zai, Yuemin Tao, Huifang Lv, Mengjia Hao, Longbiao Zhang, Xiaoyue Zhang, Yayun Xu, Yihao Zhang, Feihu Chen

**Affiliations:** 1 School of Pharmacy, Anhui Medical University, Hefei, 230032 China; 2 The Key Laboratory of Anti-Inflammatory and Immune Medicine, Ministry of Education, Anhui Medical University, Hefei, 230032 China; 3 School of Public Health, Anhui Medical University, Hefei, 230032 China; 4 Department of Health Inspection and Quarantine, School of Public Health, Anhui Medical University, Hefei, 230032 China; 5 Shenzhen Institute of Translational Medicine, Shenzhen Second People’S Hospital, The First Affiliated Hospital of Shenzhen University, Shenzhen, 518035 China

**Keywords:** Rheumatoid arthritis, Acidosis, Cytokine, AP- 1, Multi-omics

## Abstract

**Background:**

Tissue acidosis is a key characteristic of RA. It remains unclear whether acidosis promotes the formation of the complex adaptive immune landscape mainly characterized by T cell activation in RA by influencing synovial fibroblasts. This study aims to investigate the influence of acidosis on the immune microenvironment of RA by exploring the cytokine secretion and expression of co-stimulatory factors of RA synovial fibroblasts.

**Methods:**

The Bulk RNA-seq dataset (GSE89408, Normal = 23, RA = 150) was utilized for cytokine screening and the immune state assessment based on disease stage. RNA-seq was employed to investigate cytokine and co-stimulatory molecule expression following 6 h of acid stimulation, combined with Bulk RNA-seq data to evaluate contributions to RA. Human cytokine arrays were used to confirm cytokine accumulation in supernatants after 12 h of acid stimulation. Proteomics was applied to explore cellular functional states in RASFs under 6 h of acid stress, with joint RNA-seq analysis elucidating transcription factor activation. Validation of select high-throughput data was performed using qRT-PCR and immune-based assays.

**Results:**

Bulk RNA-seq and RNA-seq identified 56 differentially expressed cytokines at their intersection. Functional enrichment analysis demonstrated that acid stimulation enhanced cytokine secretion and T cell chemotaxis in RA synovial fibroblasts (RASFs). Cytokine array revealed that acid exposure increased the accumulation of growth factors (e.g., FGF, VEGF) by over twofold and promoted the expression of multiple inflammatory and chemotactic factors. Immune state analysis indicated that acid stimulation induced a complex immune landscape by upregulating co-stimulatory and antigen-presenting molecules. Proteomics showed that acid stress enhanced mitochondrial function and triggered metabolic reprogramming in RASFs. Integrated transcriptomic and proteomic analyses revealed that AP1 regulates gene expression in RASFs, with its activation further confirmed by Western blotting and immunofluorescence.

**Supplementary Information:**

The online version contains supplementary material available at 10.1186/s10020-025-01181-x.

## Introduction

Rheumatoid arthritis (RA) is a chronic systemic autoimmune disorder characterized by the presence of circulating autoantibodies, predominant synovial inflammation, structural degradation of accumulated cartilage and adjacent bone, as well as systemic inflammatory responses (Gravallese and Firestein 2023; Matteo et al. 2023; Smolen et al. 2016).The presence of a diverse array of complex cytokines in both the joint and circulatory systems is a hallmark of RA and serves as a significant contributor to its pathological progression and unfavorable prognosis (Kugler et al. 2023; Burmester et al. 2014). The utilization of monoclonal antibodies has swiftly managed the progression of the disease within a brief timeframe, and the synergistic application of multiple monoclonal antibodies alongside disease modifying anti-rheumatic drugs (DMARD) frequently offers sustained disease control for patients, potentially enabling drug discontinuation (Croft et al. 2024; Lamb and Deeks 2018). However, the recurrence of RA remains a formidable conundrum to overcome, concomitant with the excessive secretion of proinflammatory cytokines and irreversible joint impairment (Mankia and Emery 2019). A plethora of studies have manifested that the inflammatory cytokines of active RA primarily emanate from immune cells predominated by macrophages, CD4^+^T cells, and B cells (Kugler et al. 2023; Firestein 2004). Recently, the subtypes of CD8^+^ T cells have also entered the purview of researchers. GZMK^+^CD8^+^T cells featuring senescence and exhaustion possess the capacity for spontaneous cytokine secretion, maintaining the inflammatory response in RA (Burmester et al. 2014; Jonsson, et al. 2022). Nevertheless, Prior to the maturation of RA autoimmune responses, cytokine signals originating from synovial fibroblasts are essential for facilitating the recruitment and functional activation of peripheral immune cells within the joint cavity (Zhang et al. 2023; Wang et al. 2018). Furthermore, the reactivation of resident immune cells observed in RA patients following pharmacological intervention as they transition into an active disease phase has been found to correlate with cytokines secreted by synovial fibroblasts (Mankia and Emery 2019). Nevertheless, the specific characteristics of the inflammatory cytokines secreted by synovial fibroblasts remain inadequately explored. Furthermore, Recent research has indicated that Programmed cell death ligand 1 (PDL1) signaling facilitates humoral immune processes in RA (Ogishi et al. 2024). However, the source of co-stimulatory molecule signals and their influence on T cell function in RA remain insufficiently investigated.

Clinical research has demonstrated that the reduction in tissue pH is intricately associated with pathological conditions such as malignancies and tissue trauma, exemplified by stroke (Lai et al. 2024; Rastogi et al. 2023). Tissue acidosis constitutes a prominent pathological trait of RA engendered by chronic inflammation, with patients exhibiting a notable reduction in pH levels within the joint cavity, reaching as low as pH 5.0 (Cummings and Nordby 1966; Farr et al. 1985; Geborek et al. 1989). Acidosis commonly emerges as a subsequent manifestation of inflammation, Nonetheless, the potential of accumulated H^+^ to independently drive inflammatory or immune responses has not been systematically explored. In our previous study, we reported that acid-sensing ion channels (ASICs), a class of acid-sensitive ion channels activated by low extracellular pH signals, play a crucial role in the pathogenesis of RA synovial inflammation (Xu et al. 2022; Niu et al. 2020; Zai et al. 2022). Among these channels, ASIC1a functions as a rapidly activatable subunit with permeability to extracellular calcium ions. The secretion of RANTES is initiated by an increase in intracellular Ca^2+^, which subsequently modulates the activation of the transcription factor nuclear factor of activated T-cells 4(NFAT4) (Zhang et al. 2020; Bucher et al. 2020). Vascular endothelial growth factor (VEGF) has also been recognized as a cytokine released by synovial fibroblasts following the activation of ASIC1a (Qian et al. 2021). These observations suggest that tissue acidosis may play a pivotal role in sustaining and potentially enhancing the immune response. Additionally, multiple studies have demonstrated that acidosis upregulates PD-L1 expression in tumor tissues, thereby compromising anti-tumor immunity (Knopf, et al. 2023; Huntington, et al. 2022). However, the extent to which acidosis influences the expression of co-stimulatory molecules in RA remains to be elucidated.

In this study, we focused on analyzing the dynamic expression and release of cytokines in RA synovial fibroblasts (RASF) in response to acidic stimulation, as well as the functional state of RASF. Our findings indicate that RASF exposed to low pH stress significantly increased the secretion of chemokines and growth factors, thereby enhancing the recruitment and activation of immune cells. Additionally, acidosis modulated the expression levels of various co-stimulatory molecules. The altered expression of cytokines and co-stimulatory factors in RASF may influence the immune microenvironment within the joint cavity of RA patients. Furthermore, we observed that acute low pH stress enhanced oxidative phosphorylation and mitochondrial function in RASF and effectively activated the transcriptional network centered on Activator Protein 1 (AP- 1), which regulated gene expression following acidic stimulation.

## Methods

### Public datasets

Synovium Bulk RNA-seq dataset of 23 normal subjects and 150 RA patients (including 57 with early RA and 93 with established RA) were obtained from the GEO public database (GSE89408, www.ncbi.nlm.nih.gov/geo/). The edgeR v4.2.2 package in R was used for differential analysis of Raw read counts. *Homo_sapiens GRCh38.112. GTF. Gz* annotated datasets were used to calculate Fragments Per Kilobase Per Million mapped Fragments (FPKM). The demographic features of the patients in the samples of GSE89408 involved in this study is presented in Table 1.

**Table 1:**
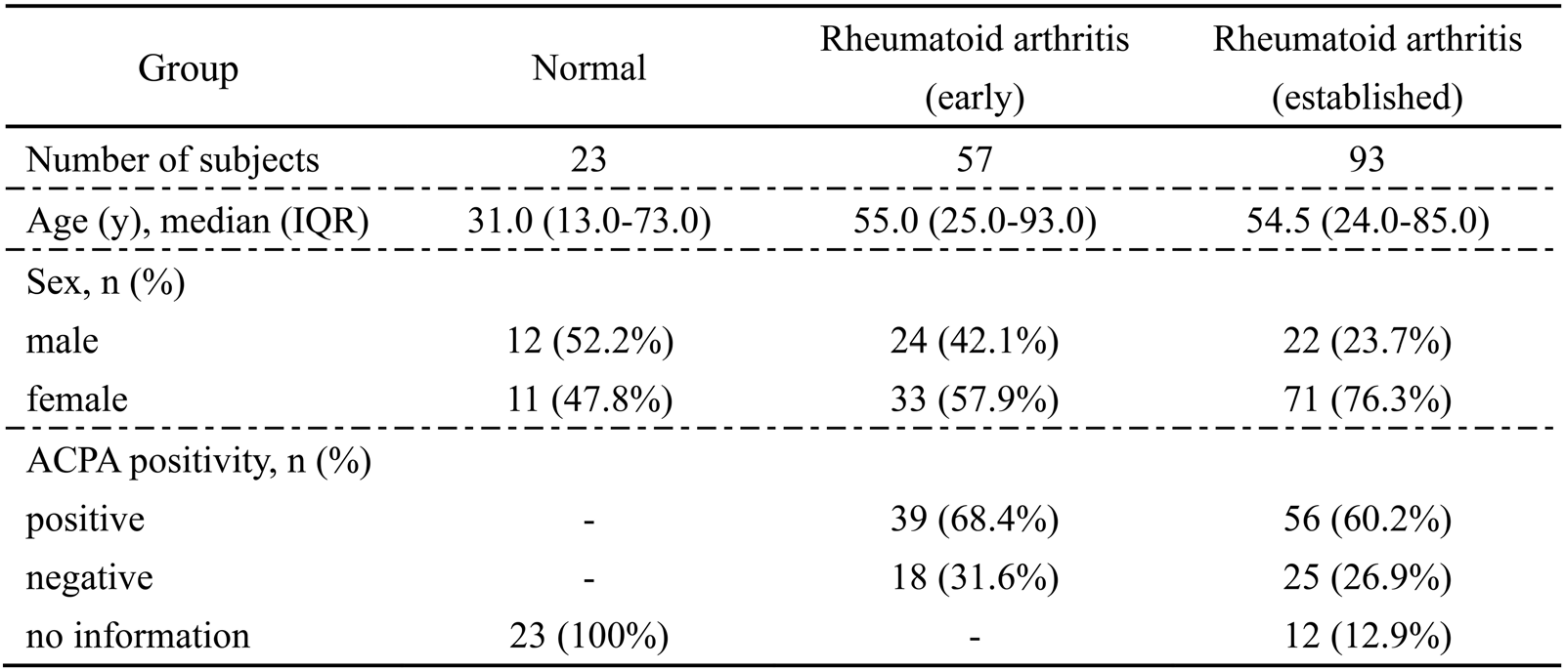
Demographic features of subjects in GSE89408 dataset

*ACPA *Anti-citrullinated peptide antibodies

### Isolation, culture and treatment of synovial fibroblasts

This study has been reviewed and approved by the Human Research Ethics Committee of Anhui Medical University (20,210,331, Hefei, China), and has obtained the patient's informed consent. RA knee synovial tissues were obtained from 18 RA patients (6 men and 12 women) who underwent knee replacement between 2021 and 2024. Primary RASFs was isolated from the synovial tissue of knee joints in patients with RA using a previously established method (Zhang et al. 2020). The cells were cultured in Dulbecco's modified eagle medium (DMEM)/high-glucose medium (11,965,092, Gibco, USA), supplemented with 20% bovine serum (10099141 C, Gibco, USA), along with 100 IU/mL penicillin and 100 μg/mL streptomycin. The cultures were maintained in a cell incubator (HERA Cell 150I, Thermo Fisher Scientific, USA) at 37 °C under an atmosphere of 5% CO2. All experiments were performed using synoviocyte cultures from 4 to 7 th passages.

### RNA sequencing and data analysis

The RASF was cultured for 6 h in a medium with the pH adjusted to 6.0 using concentrated hydrochloric acid. Total RNA was extracted utilizing TRIzol reagent (15,596,026, Thermo Fisher Scientific, USA). The purity of RNA was assessed using a NanoDrop 2000 spectrophotometer, while its integrity was evaluated with an Agilent 2100 bioanalyzer. Reverse transcription of RNA and library construction were performed according to the manufacturer's instructions employing the NEBNext^®^ Ultra™ II RNA Library Prep Kit for Illumina (E7770, NEB, USA). The cDNA library quantification was conducted using the QuantiFluor^®^ dsDNA system (E2670, Promega, USA). RNA sequencing was executed on the NovaSeq™ 6000 system (Illumina, USA) utilizing the NovaSeq 6000 S Prime Reagent kit with paired-end reads of 150 nucleotides each. Cutadapt version 1.15 software facilitated trimming and quality control of the original paired-end reads. Selected reads were aligned against the reference genome (Human Genome Assembly GRCh38) using hisat2 version 2.0.5 localization model respectively. Differentially expressed genes (DEGs) were analyzed through DESeq2 program with criteria set at |fold change|≥ 1.5 and P value < 0.05. Raw read counts for each gene were obtained and compared using HTSeq version 0.9.1 software to calculate FPKM for subsequent analysis.

### Quantitative real-time polymerase chain reaction (qRT-PCR)

RASF was cultivated for 12 h in the acidified medium with the pH adjusted to 6.0 by concentrated hydrochloric acid, the acidified medium containing 100 nM of the specific inhibitor PcTx- 1 for ASIC1a, the acidified medium containing 1 mM of the extracellular calcium ion chelator EGTA, and the acidified medium containing 10 μM of the intracellular calcium ion chelator BAPTA-AM. Total RNA was extracted from samples using the TRIzol Reagent (AG Scientific, China) following the manufacturer's protocol. The extracted RNA was reverse transcribed into complementary DNA (cDNA) using the Evo M-MLV RT Premix for qPCR (AG Scientific, China). Quantitative real-time polymerase chain reaction (qPCR) analysis was performed using the SYBR Green Premix Pro Taq HS qPCR kit (AG Scientific, China). ACTB was used as an internal control for and mRNAs. Primer sequences utilized in this study are listed in Table 2.

**Table 2:**
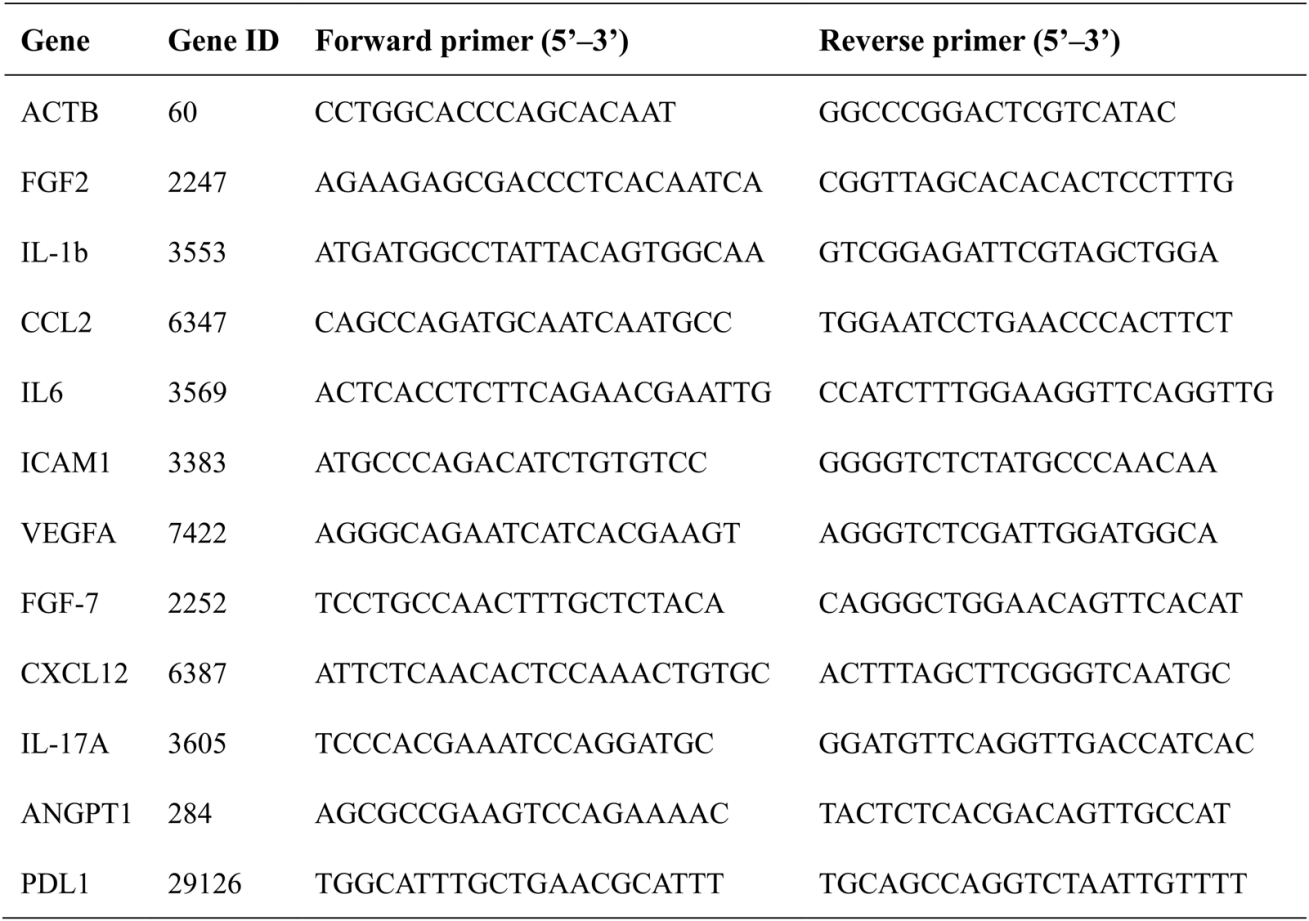
Primer sequences for qRT-PCR

### Human cytokine array (HCA)

Cells were treated in DMEM/high glucose medium containing 1% FBS for 12 h. After 12 h, the supernatants from each treatment group were collected. A human cytokine array kit (R&D Systems, ARY022B, USA) was used to quantify cytokines in the cell supernatants. Briefly, 2 mL of 1 × blocking buffer was added to each well of a 4-well multi-well plate, and nitrocellulose membranes pre-coupled with cytokine antibodies were placed into individual wells and blocked at room temperature for 1 h. The blocking buffer was then removed, and the samples were diluted to 1.5 mL with 1 × blocking buffer. The diluted samples were added to the multi-well plate and incubated overnight on a shaker at 2–8 °C. Each membrane was subsequently removed and placed into a plastic container with 20 mL of 1X wash buffer. The 4-well multi-dish was rinsed and dried. Each membrane was washed twice with 1X wash buffer on a shaker for 10 min per wash. A 20 μL detection antibody mixture was added to 1.5 mL of blocking buffer, and the diluted antibody mixture was added to each well of the 4-well multi-well plate at 1.5 mL per well. The membranes were then placed into the 4-well multi-well plate and incubated on a shaker for 1 h, followed by another wash. Next, 2 mL of 1X Streptavidin-HRP was added to each well of the 4-well multi-well plate, and the washed membranes were placed into the wells and incubated on a shaker at room temperature for 30 min. After another wash, the membranes were incubated with the developing reagent for 1 min, and signals were captured using a chemiluminescence imaging system. The array distribution and information of 105 cytokines is shown in the Supplementary Fig. 3E and Table 3.

**Table 3:**
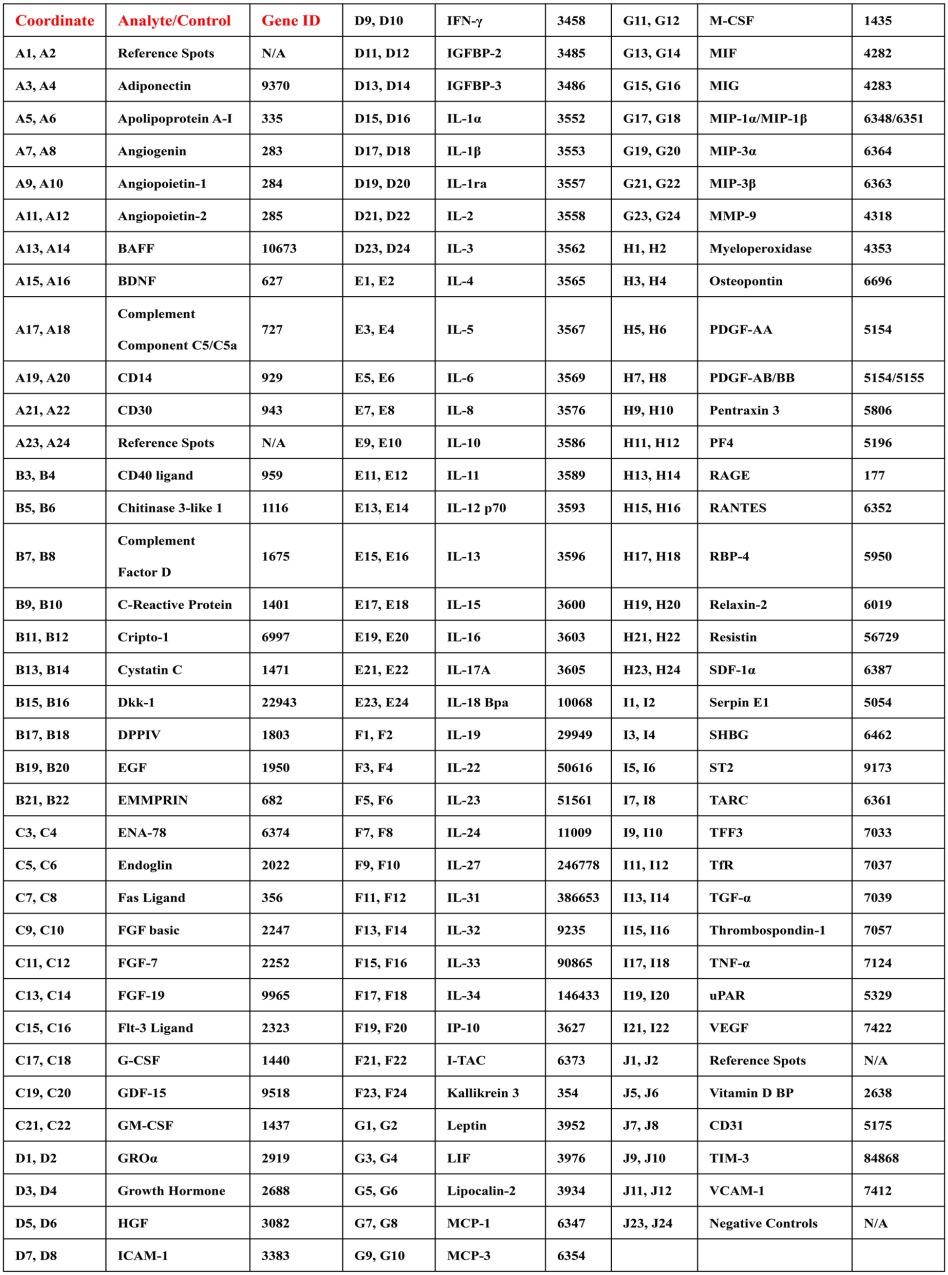
Information of HCA plates

### Label-free quantitative proteomics

Cells was cultured in a medium with concentrated hydrochloric acid adjusted to pH 6.0 for 6 h, and the total protein was extracted using a micro/total protease digestion kit (QLBIO MAGicomicomicS-MMB8X). 20 μL of protein was incorporated into the octa housing MMB beads and subjected to incubation at 37 °C for 30 min. Subsequently, 45 μL of binding buffer was introduced and underwent equilibration at room temperature for 15 min. Post-incubation, the supernatant was disposed of, and the MMB beads were rinsed thrice with the washing buffer. The magnetic beads were then re-suspended in 20 μL of enzymatic cleavage solution and underwent incubation at 37 °C for over four hours. After this incubation period, 5 μL of quenching buffer was added to terminate the enzymatic digestion, subsequent to which the samples were lyophilized.

The mobile phase comprised liquid A (100% water, 0.1% formic acid) and liquid B (80% acetonitrile, 0.1% formic acid), which were both meticulously prepared. The lyophilized powder was reconstituted using 10 µL of solution A and subsequently centrifuged at 14,000 g for 20 min at 4 °C. Subsequently, a 1 µg sample from the supernatant was injected for liquid chromatographic analysis. The elution time is adjusted based on the proportion of mobile phase B. The entire process comprises the following steps: 8% B at 0 min, 12% B at 5 min, 35% B at 35 min, 40% B at 44 min, 95% B at 45 min, and maintaining 95% B from 45 to 60 min. For mass spectrometry analysis, a QExactive HF-X mass spectrometer equipped with a Nanospray Flex™ (NSI) ion source was employed; the ion spray voltage was set at 2.2 kV and the temperature of the ion transport tube was maintained at 320 °C. The data-dependent acquisition mode was implemented for mass spectrum acquisition, encompassing a full scanning range from m/z 350 to m/z 1500. The primary mass spectrum resolution was established at 120,000 (at m/z 200), with an automatic gain control (AGC) target set to 3 × 10^^^6 and a maximum injection time for the C-trap fixed at 80 ms. Parent ions demonstrating the top 40 ionic strengths during full scans were selected for fragmentation via high-energy collision-induced dissociation (HCD), followed by secondary mass spectrometric detection. The resolution for secondary mass spectrometry was configured at 15,000 (at m/z 200), with an AGC target of 5 × 10^^^4 and a maximum injection time limited to 45 ms; additionally, the peptide fragment fragmentation collision energy was adjusted to approximately 27%. Raw data files (.raw) were generated as part of this procedure.

### Clustering by fuzzy C-means (FCM) algorithm

The gene clustering analysis was carried out in accordance with expression patterns and implemented through the ClusterGVis v0.1.2 package in R, the clustering method was mfuzz (https://github.com/junjunlab/ClusterGVis). Subsequently, the enricher function was employed for the functional categorization of clusters, leveraging representative subsets from Kyoto Encyclopedia of Genes and Genomes (KEGG) and Gene Ontology (GO).

### Identification of enriched gene pathways

The enriched gene pathways were discerned via pre-sequenced Gene Set Enrichment Analysis (GSEA), executed with the R package fgsea version 1.30.0 leveraging statistically significant gene arrangements. Grounded on differential expression profiling, the gene rank was computed by the formula -log (P value) × log (fold change). To appraise the enrichment status of pathways within the cluster, subsets from msigdbr v.7.5.1 encompassing GO, KEGG, REACTOME, Pathway Interaction Database (PID), and Transcription Factor Target (TFT) pathways were utilized. We assert that it is of paramount significance to implement Benjamini–Hochberg correction for pathways with P values < 0.25.

### Gene set variation analysis

Functional enrichment analysis of FPKM values utilizing the REACTOME gene set was conducted using the GSVA v1.52.3 package in R. The scores for all pathways are manifested through heat maps and bar charts.

### Western blotting

Total proteins were extracted from synovial fibroblasts at different time points using RIPA (Beyotime, China). The lysate was mixed with the loading buffer, underwent SDS-PAGE (80 V for 30 min, then 120 V for 60 min), and was electrotransferred to a PVDF membrane (Millipore Corp., Billerica, MA, USA). The PVDF membrane was blocked with Tween 20-containing TBS-T and 5% skim milk for 2 h. Then, it was incubated overnight at 4 °C with anti-JUN antibodies (Abcam, ab280089, USA), anti-FOS antibodies (Absin, abs149671, China), anti-JUNB antibodies (Affinity, #AF6198, China), anti-FOSB antibodies (Affinity, #AF5010, China), anti-JUND antibodies (Affinity, #AF6200, China), anti-GAPDH antibodies (Proteintech, 60,004–1-Ig, China). The next day, after washing, goat anti-mouse or anti-rabbit antibody coupled with HRP was incubated for 1 h. Finally, the membrane was imaged using an ECL-plus kit (Thermo Fisher Scientific), and density analysis was done by ImageJ software.

### Building of protein interaction network

The protein–protein interaction (PPI) network of the AP1 subunit was analyzed through the utilization of the STRING database (https://cn.string-db.org/). The analysis parameters were set as follows: Full STRING network. Evidence; medium confidence level (0.400); a maximum of 50 interacting proteins. Molecular module and pathway enrichment analyses were carried out by employing the cytoHubba plugin within Cytoscape v3.10.1 software. Based on the GO, KEGG and TFT databases, pathway enrichment analysis for the AP1 protein interactions was executed using g: Profiler (https://biit.cs.ut.ee/gprofiler/gost).

### Multicolor immunofluorescence

Six paraffin-embedded synovial tissue sections (OA = 3, RA = 3) were screened from published studies (Zhang et al. 2020; Qian et al. 2021). The sections were initially dewaxed with xylene and subsequently hydrated in sequence with ethanol of varying concentrations. Subsequently, antigen retrieval and blocking processes were performed. RASF were seeded into glass-bottom culture dishes, and after reaching an appropriate density, they were treated according to the experimental groups. The cells were then fixed with paraformaldehyde, and 0.1% Triton X- 100 was used for permeabilization if needed, followed by blocking with 5% BSA. Multiplex fluorescence staining was performed using the Sextuple-Fluorescence Immunohistochemical Mouse/Rabbit Kit (pH 9.0) (Immunoway, RS0039, USA). Briefly, different secondary antibodies were labeled with distinct amplification systems based on fluorescent tyramide signals. An ethylene diamine tetraacetic acid (EDTA)-based antigen extraction method was applied between the two rounds of tyramide signal amplification to remove antibodies from the previous step. DAPI was used as a nuclear stain. Samples were analyzed using a panoramic tissue quantitative analysis system (Zeiss, Germany). Antibodies used in this experiment included anti-JUN antibody (Abcam, ab280089, USA), anti-FOS antibody (Absin, abs149671, China), anti-JUNB antibody (Affinity, #AF6198, China), anti-FOSB antibody (Affinity, #AF5010, China), anti-JUND antibody (Affinity, #AF6200, China), anti-HLA-A antibody (bio-swamp, RMAB49423, China), anti-HLA-B antibody (Abcam, ab168436, USA), anti-HLA-C antibody (Bioss, bs- 10251R, USA), anti-PDL1 antibody (Absin, abs136046, China), anti-PDPN antibody (Proteintech, 11,629–1-AP, China), anti-CDH11 antibody (Affinity, #DF3523, China), anti-CD3 antibody (Proteintech, 17,617–1-AP, China), anti-CD4 antibody (Absin, abs120100, China), and anti-CD8 antibody (Absin, abs120101, China).

### Statistical analysis

Results are shown as mean with 95% confidence intervals. A one-way analysis of variance (ANOVA) was employed to compare means across three or more groups. One-way ANOVA and Dunnett's multiple comparison test were employed to assess the significance of individual differences between the in vitro control group and the group exposed to acid stimulation. All statistical analyses were conducted using SPSS version 23.0 (SPSS Inc., Chicago, USA) and GraphPad Prism version 8.0 (GraphPad Software Inc., Boston, USA). P values < 0.05 were considered statistically significant; specifically, *P < 0.05 indicates significance, **P < 0.01 denotes a higher level of significance, * * * P < 0.001 reflects very high significance;"ns"indicates no statistical significance.

## Results

### RA synovium exhibited elevated cytokine secretion and regulation of T cell activation.

**Fig. 1:**
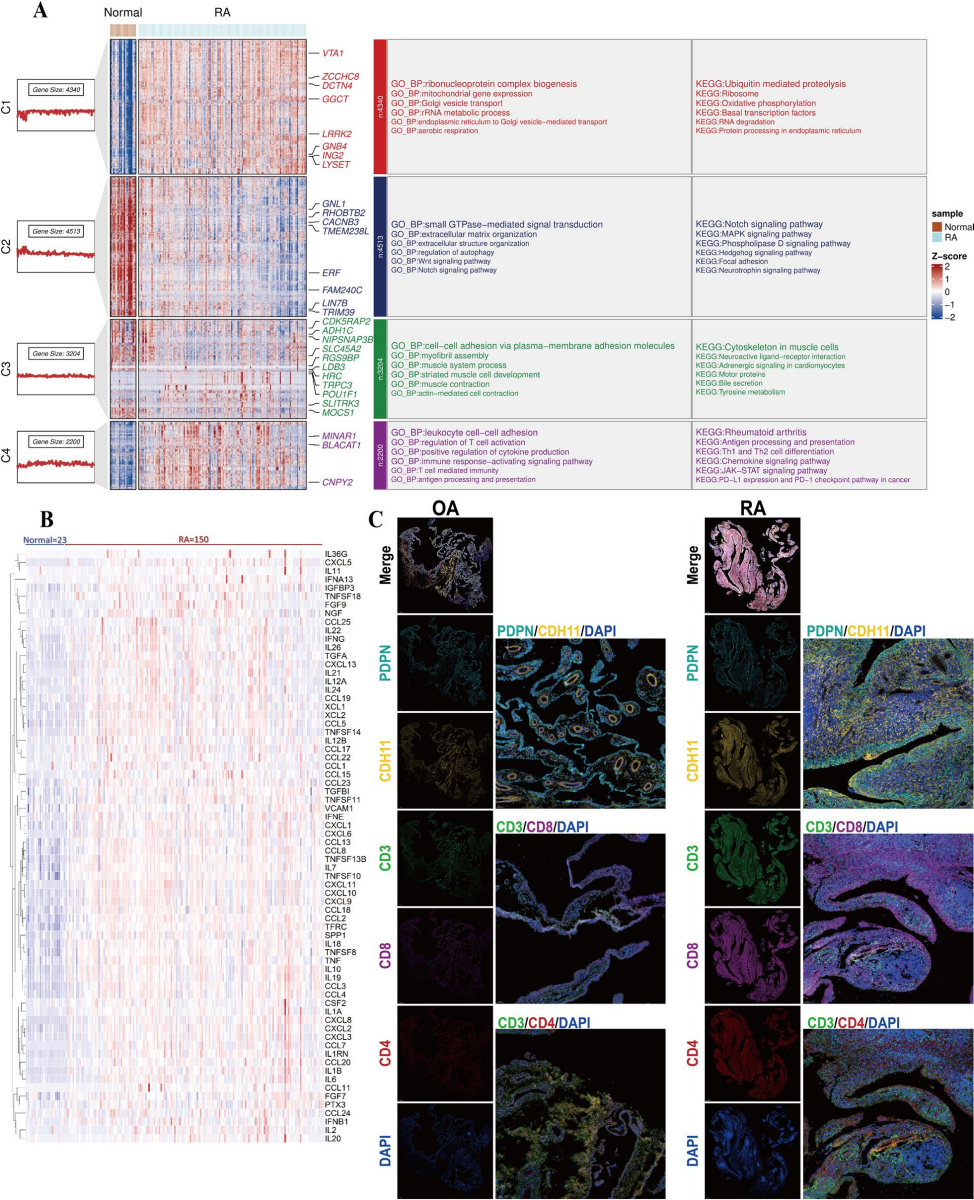
RA synovial tissue is typified by elevated cytokine secretion and enrichment of T cells. **A** The GSE89408 dataset (normal = 23, RA = 150) was clustered in accordance with the expression pattern, and the cluster was enriched based on KEGG database and GO database.** B** Heatmap revealed the cytokine expression profiles with significant differences in the GSE89408 dataset. **C** The distribution of T cells in OA synovium and RA synovium was characterized through multiple immunofluorescence (OA = 3, RA = 3), Scale bar = 500 μm (Overall landscape) and 100 μm (Selected landscapes)

Synovial tissue emerged as the prime locus where RA manifested pathological traits (Pandey and Bhutani 2024). Therefore, we initially focused on evaluating cytokine expression and T cell status in RA synovial tissue. The annotation of dataset GSE89408 reveals RNA-seq data from 28 normal subjects, 22 osteoarthritis (OA) patients, 10 arthralgia patients, 57 early RA patients, 95 established RA patients, and 6 undifferentiated arthritis samples. Further inspecting the original counts, we chose samples from 23 normal subjects, 57 early RA patients, and 93 established RA patients (The missing sample data for analysis were absent from the original counts). Multiple volcano plots were employed to illustrate the differential gene expression in the GSE89408 dataset (Supplementary Fig. 1 A). Based on the FCM algorithm, the heat map of RNA data from normal subjects and RA patients was displayed after clustering, and the cluster genes were annotated with GO and KEGG (Fig. 1A). The results showed that the GO enrichment of RA tissue in C1 cluster showed significant enhancement of ribosome, mitochondria, endoplasmic reticulum and other organelles. KEGG enrichment also supports the enhancement of intracellular RNA and protein anabolism and mitochondrial oxidative phosphorylation in RA synovium. The C4 cluster shows a significant enrichment trend in cytokine secretion and the regulation of immune cells, especially T cells. Moreover, it has significant enrichment in antigen extraction, T cell differentiation, cytokine signaling pathways, the JAK-STAT pathway, and PDL1-PD1 co-stimulatory signals. The GSEA enrichment analysis substantiates the preceding result (Supplementary Fig. 1B–D). Cytokines exhibiting significant differences in GSE89408 were identified, with a screening threshold set at P < 0.05 (Fig. 1B). To assess the status of T cells in RA synovial tissue, multi-immunofluorescence of T cell labeling in synovial tissue revealed that RA synovial tissue exhibited more pronounced inflammatory infiltration, and CD3^+^CD8^+^ T cells were more extensively distributed than CD3^+^CD4^+^ T cells (Fig. 1C).

### Extracellular acidification markedly enhanced cytokine expression in RASF

**Fig. 2:**
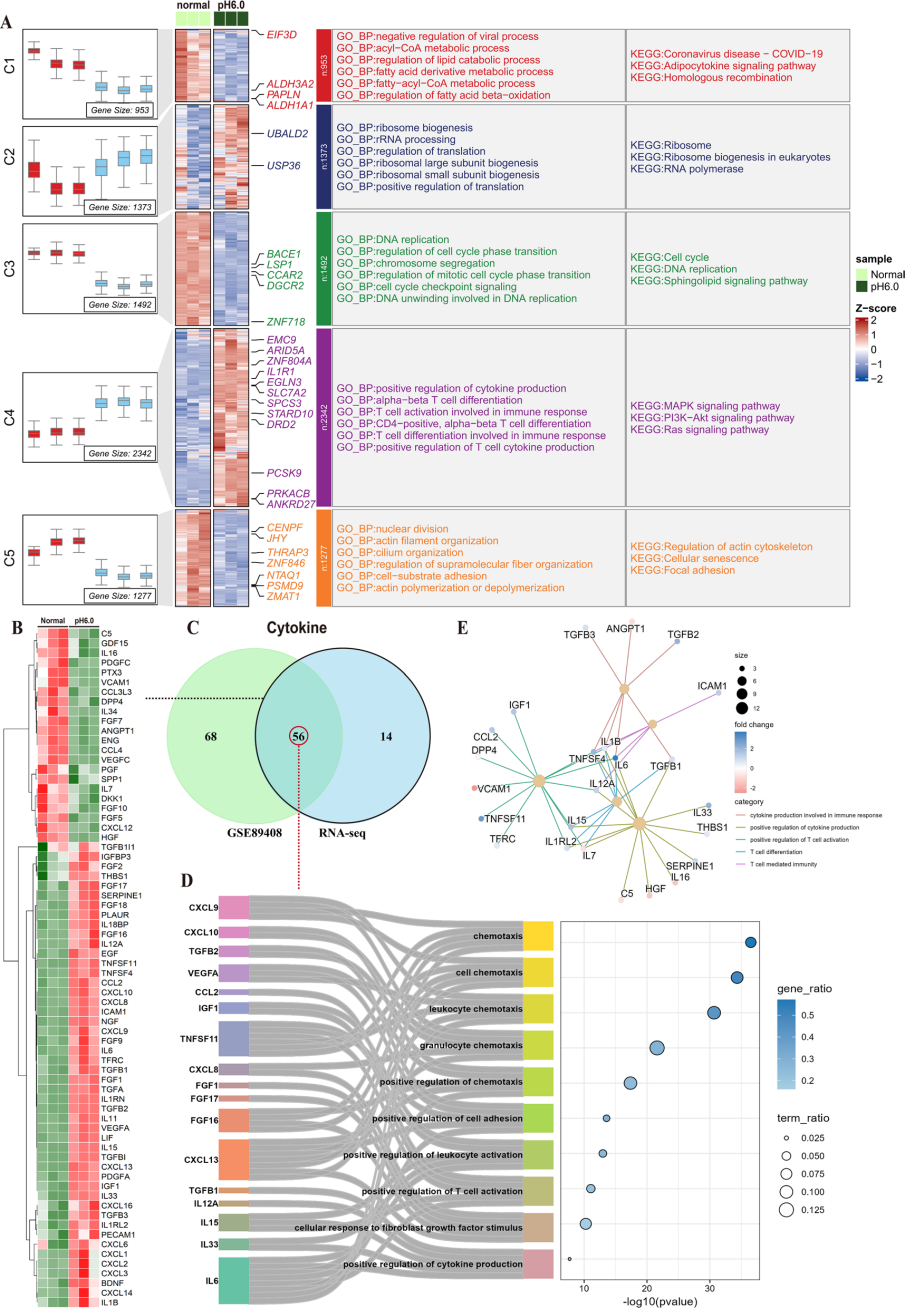
Effects of acid stimulation on immune activation and cytokine secretion of RASFs. RASFs were treated for 6 h in complete media adjusted to pH6.0 with hydrochloric acid (HCl), followed by RNA sequencing.** A** RNA-seq expression profiles were clustered according to FCM algorithm, and the clusters were enriched based on KEGG database and GO database.** B** Heatmap revealed the expression profile of cytokines with significant differences in RNA-seq. **C** Venn diagram showed the intersection of cytokines between the GSE89408 dataset and RNA-seq. **D** Enrichment analysis of GO pathways of the intersecting cytokines, and the contribution of cytokines is presented via Sankey plots. **E** The association between cytokines and the immune activation pathways of T cells was identified by network

Previous investigations have manifested that acidosis augments the secretion of RANTES, IL- 6, VEGF, and other cytokines in RASF (Zhang et al. 2020; Qian et al. 2021). With the aim of probing whether synovial fibroblasts have the capability to function as the prominent secretory entity of copious cytokines in RASF and whether the low extracellular pH potentiates the maturation of intricate immune states, RASF stimulated with pH 6.0 were quantitatively assayed using RNA-seq. The differentially expressed genes in RNA-seq are presented through a volcano plot (Supplementary Fig. 2 A). Based on the FCM algorithm, RNA expression was classified into five disparate clusters. Moreover, GO and KEGG enrichment analyses manifested a significant upregulation of genes implicated in ribosome biosynthesis, protein translation, cytokine secretion, and T-cell-mediated immune activation in RASF (Fig. 2A). The enrichment of the T-cell receptor signaling pathway and the PD-L1/PD- 1 signaling pathway was identified through KEGG database analysis. (Supplementary Fig. 2B). GSEA enrichment showed significant activation of pathways related to cytokine and T cell activation (Supplementary Fig. 2 C-D). The GSVA enrichment analysis revealed significant upregulation of pathways associated with T cell activation. Notably, histone methylation and chromatin aggregation exhibited inhibitory states Supplementary Fig. 2E). Subsequently, a total of 70 cytokines exhibiting significant differences, potentially implicated in immune regulation, cell chemotaxis, and other biological functions, were identified (Fig. 2B). These cytokines were compared with those showing significant differences in the GSE89408 dataset, and 56 intersections of cytokines were selected (Fig. 2C). Sankey enrichment maps show that cytokines at these intersections mainly regulate lymphocyte chemotaxis and activation as well as fibroblast growth and cell adhesion. IL6 and CXCL13 have the most extensive biological functions and are widely involved in these regulations (Fig. 2D). The construction of the network map for related biological functions like immune activation by these cytokines and T cells proved that IL6 and CXCL13 are central molecules in these functions (Fig. 2E). These observations imply that the marked increase in cytokine secretion upon extracellular acidic stimulation induce T cell activation and immune modulation.

### Acidosis sustains and amplifies the secretory phenotype of RASF

**Fig. 3:**
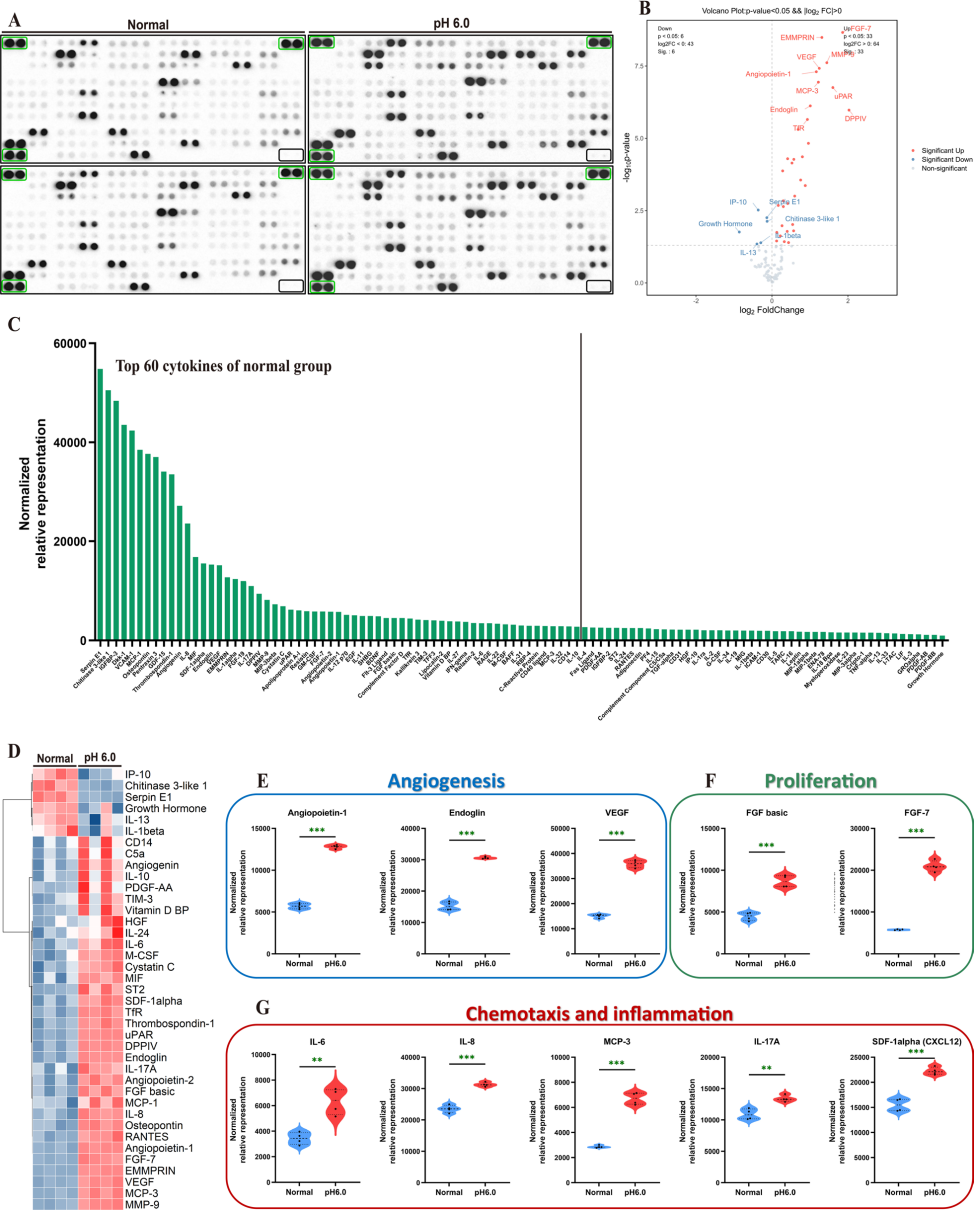
Extracellular acidification stimulation promotes the secretion of cytokines in RASF. **A** The expression of cytokines in the culture supernatant was determined by HCA. **B** The volcano plot demonstrated the differentially expressed cytokines in HCA.** C** Relative quantification and ranking of cytokine expression in the culture supernatants of RASFs treated with pH7.4 culture medium.** D** Heatmap presented the differentially expressed cytokines in HCA. **E**–**G** The statistical analysis of the expression quantities of cytokines was performed in accordance with the three modules of angiogenesis, proliferation, chemotaxis, and inflammation. Data are presented as the mean ± SD. Statistical significance was determined by one-way ANOVA followed by Dunnett’s multiple comparison test: **P < 0.01, ***P < 0.001, ns: not significant

**Fig. 4:**
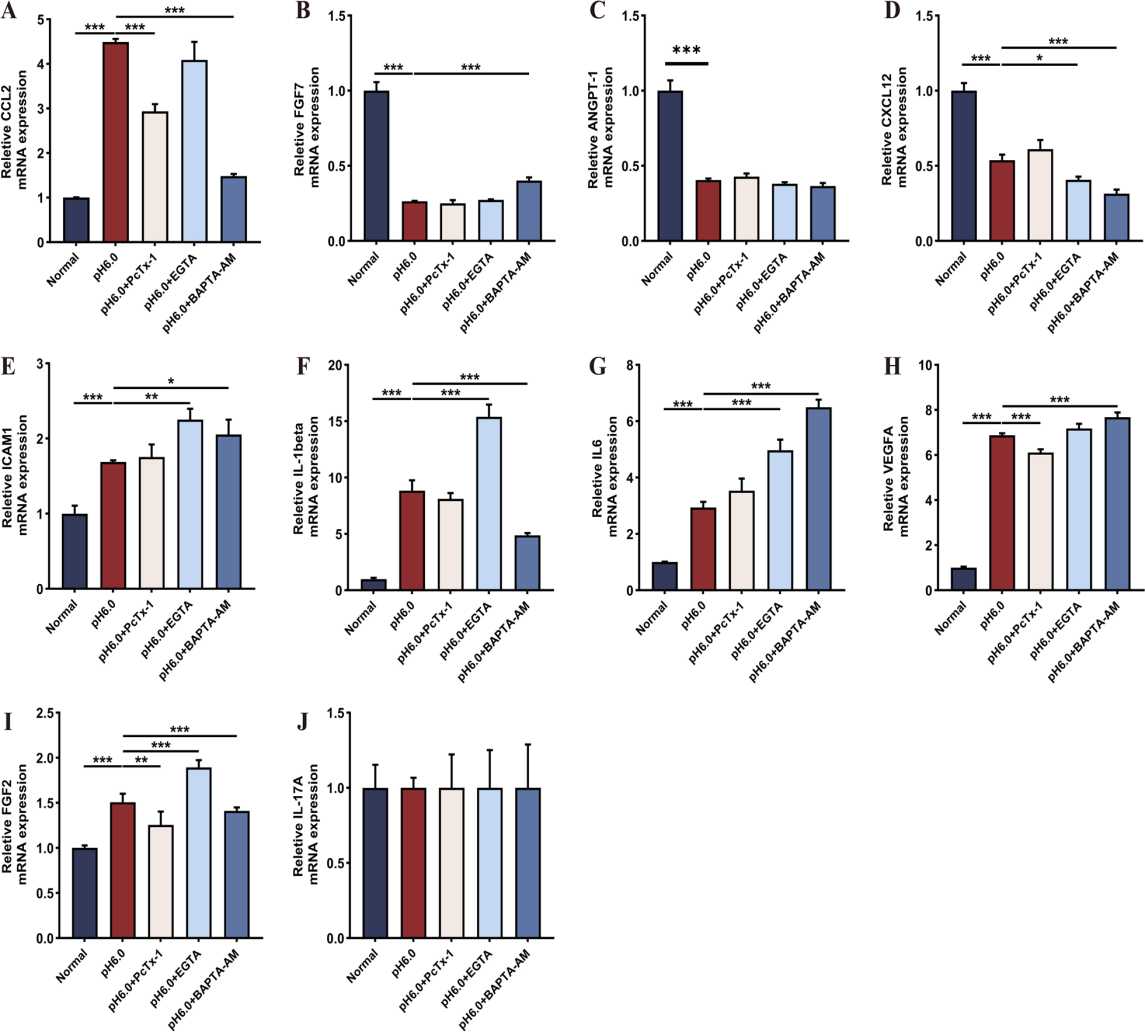
Acidosis regulates the expression of cytokine genes in RASF. The expression of various cytokines under acid stimulation was verified through the utilization of 100 nM PcTx- 1, 1 mM EGTA, 10 μM BAPTA-AM. **A-J** Quantification of gene expression levels of CCL2, FGF7, ANGPT- 1, CXCL12, ICAM1, IL- 1β, IL- 6, VEGFA, FGF2, and IL- 17 A in acidification-stimulated RASF treated with PcTx- 1, EGTA, and BAPTA-AM was performed using qRT-PCR. Data are presented as the mean ± SD from three independent experiments. Statistical significance was determined by one-way ANOVA followed by Dunnett’s multiple comparison test: *P < 0.05, **P < 0.01, ***P < 0.001, ns: not significant

Antibody microarray technology was used to verify cytokine secretion in this study (Fig. 3A). After confirming no significant difference in the quantification of Negative Controls and Reference Spots signals of each array as negative and positive controls, we quantified each cytokine based on the location information in the instructions (Supplementary Fig. 3 A). To elucidate the impact of RA background on the secretion phenotype of synovial fibroblasts, we first quantified and visualized the cytokine levels in the control group using histograms (Fig. 3C). Subsequently, among the 105 cytokines encompassed in the array, the top 60 cytokines demonstrating the most elevated baseline values within the control group were selected for functional enrichment analysis. The outcomes revealed that they predominantly modulated biological processes such as lymphocyte chemotaxis and proliferation (Supplementary Fig. 3B). Cytokine differential expression analysis demonstrated that extracellular acidification significantly enhanced the secretion of cytokines in RASF, including FGF- 7, EMMPRIN, MMP9 and VEGF (Fig. 3B). The GOBP enrichment revealed that up-regulated cytokines enhanced the pathways of lymphocyte chemotaxis, migration, and endothelial cell proliferation and the GSEA enrichment analysis concurs with the prior outcomes (Supplementary Fig. 3 C-D). The differentially expressed cytokines were presented through heat maps (Fig. 3D). Subsequently, we delineated the expression profiles of certain cytokines within three modules: angiogenesis, cellular proliferation, and chemotaxis (Fig. 3E–G). Although our previous research has established the critical role of ASIC1a-mediated calcium influx in response to extracellular acidification. qRT-PCR revealed that prolonged acidification stimulation (12 h) significantly reduced the gene expression of FGF7, ANGPT- 1, and CXCL12. In contrast, IL- 17 A showed insensitivity to external stimuli. Notably, CCL2, ICAM1, IL- 1beta, IL6, VEGF, and FGF2 maintained high expression levels under acidification conditions. However, the inhibitory effects of ASIC1a antagonists and calcium chelators on acidification-induced responses were inconsistent, with only CCL2 retaining sensitivity to PcTx- 1 and BAPTA-AM. These findings suggest that extracellular acidification may sustain the inflammatory secretory phenotype of RASF through mechanisms independent of ASIC1a and Ca^2+^ signaling. (Fig. 4A–J). These results showed that extracellular acid stimulation did not significantly change the secretion phenotype of synovial fibroblasts in RA, but maintained and enhanced the secretion intensity of most cytokines, promoting the chemotaxis and activation of fibroblasts on lymphocytes.

### Extracellular acid stimulation modulates T cell activation in the immune development of early RA

**Fig. 5:**
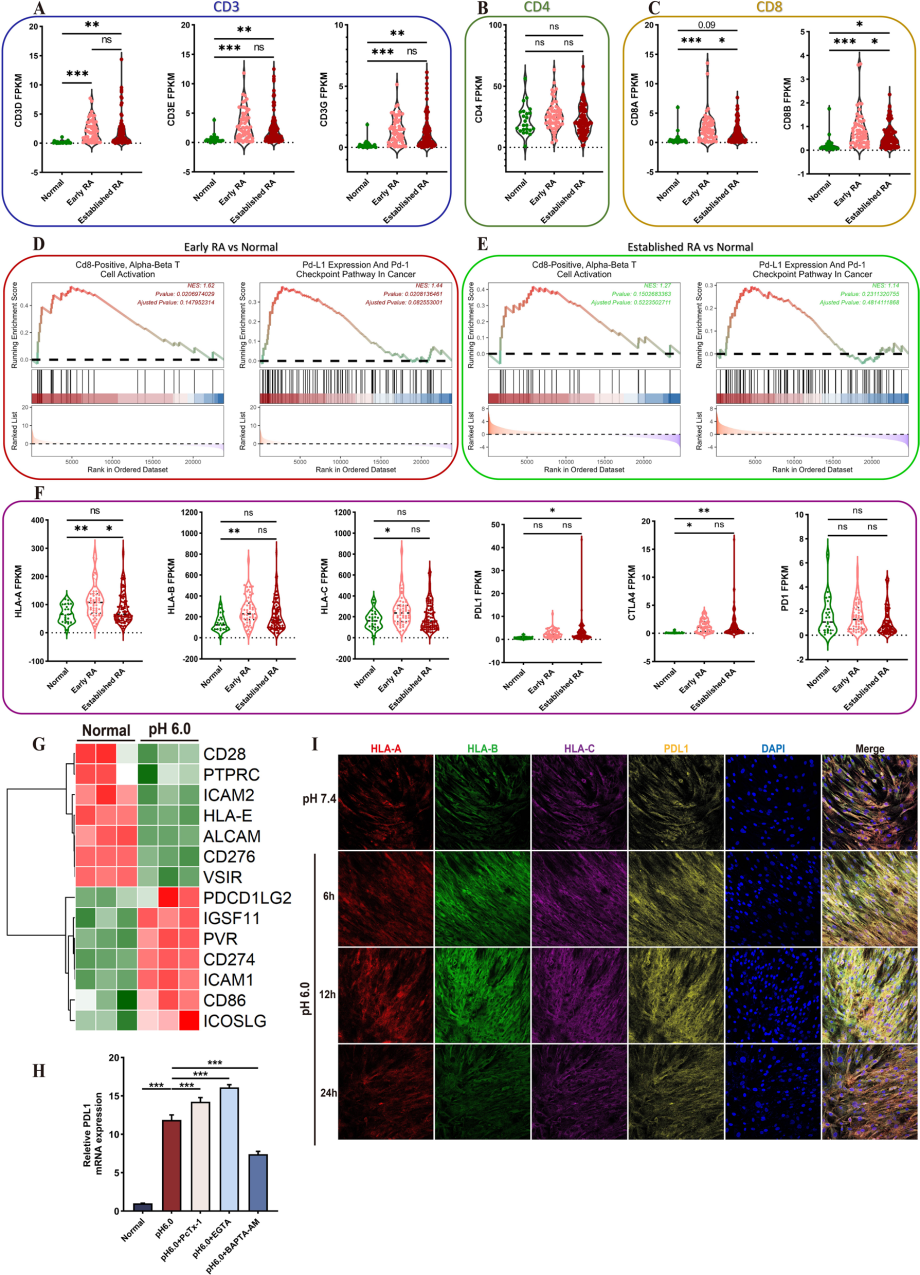
Extracellular acidification regulates the progression of T-cell immunity through the expression of co-stimulatory factors in RASF. **A-C** The alterations in the expression of T cell markers CD3, CD4 and CD8 in the course of RA progression. **D-E** The alterations in the status of CD8^+^T cell activation pathways and PDL1 pathways in early RA and established RA in comparison with the normal group. **F** The alterations in the expression of antigen presenting molecules HLA-A, HLA-B, HLA-C and co-stimulatory molecules PDL1, CTLA4, PD1 in the course of RA progression. **G** Heatmap presented the differentially expressed co-stimulatory molecules in RNA-seq. **H** Quantification of gene expression levels of PDL1 in acidification-stimulated RASF treated with 100 nM PcTx- 1, 1 mM EGTA, and 10 μM BAPTA-AM was performed using qRT-PCR. **I** Multiplex immunofluorescence presented the expression and localization of antigen-presenting molecules HLA-A, HLA-B, HLA-C and co-stimulatory molecule PDL1 in RASFs (n = 3). Scale bar = 50 μm, magnification = 200x. Data are presented as the mean ± SD from three independent experiments. Statistical significance was determined by one-way ANOVA followed by Dunnett’s multiple comparison test: **P < 0.01, ***P < 0.001, ns: not significant

Our findings indicate that acidification stimulation is directly linked to the secretion of various chemokines and inflammatory mediators, which modulate T cell activation in RA. Research has demonstrated an enrichment of CD8^+^ T cells in RA (Zhang et al. 2019). However, these CD8^+^ T cells exhibit a loss of cytotoxic functionality due to alterations in granzyme phenotypes, with the secretion of pro-inflammatory factors being the predominant characteristic, which indicates that CD8^+^ T cells might undergo phenotypic alterations in RA (Jonsson, et al. 2022). Therefore, we investigated the expression of T-cell surface antigens in each sample of the dataset GSE89408. Results showed that the expression of CD3 subunits of mature T cell antigen significantly increased in both early RA and established RA. But the expression of CD4 antigen had no significant difference among normal people, early RA and established RA. Conversely, CD8 antigen expression was significant in early RA and decreased in established RA compared with early RA (Fig. 5A–C). Early RA and established RA were respectively enriched with normal samples through GSEA. Compared with the normal control, CD8^+^ T cell activation and the PDL1 and PD1 pathways in early RA exhibited significant positive enrichment. However, the established RA showed no significant significance in the enrichment results of these pathways compared with normal samples (Fig. 5D, E). The two paramount steps in the activation of CD8^+^ T cells are the binding of antigen presenting molecules to T cell receptor (TCR) and the subsequent binding of co-stimulatory molecules. It was determined that the expression of the classical major histocompatibility complex (MHC) I receptor was conspicuously augmented in early RA, while only HLA-A in established RA manifested significant disparities when compared with the normal control. PDL1 manifested an escalating expression pattern from early RA to established RA, and only in established RA was a significant disparity observed when compared with the normal group. The expression of CTLA4 also exhibited an upward trend, and a significant difference was noted between early RA and established RA in contrast to the normal group. PD1 manifested a decline in expression from normal to established RA, though these trends did not exhibit significant variances (Fig. 5F). To determine whether extracellular acidification can modulate the co-stimulatory signaling in synovial fibroblasts, we ascertained the expression of significantly disparate co-stimulatory molecules (Fig. 5G). The result of qRT-PCR demonstrated that the expression of PDL1 was prominently augmented by acid stimulation and was reversed under the effect of BAPTA-AM (Fig. 5H). Multiple fluorescence staining manifested that acid stimulation conspicuously augmented the PDL1 expression of RASF with time-series, and MHC I also exhibited increased expression induced by acid stimulation (Fig. 5I). These results suggest that extracellular acids promote the antigen presentation process and regulate the expression of co-stimulatory molecules, and these processes modulate the activation of T cells and the final landscape of the immune response.

### Acid stimulation stress enhanced the mitochondrial function of RASF

**Fig. 6:**
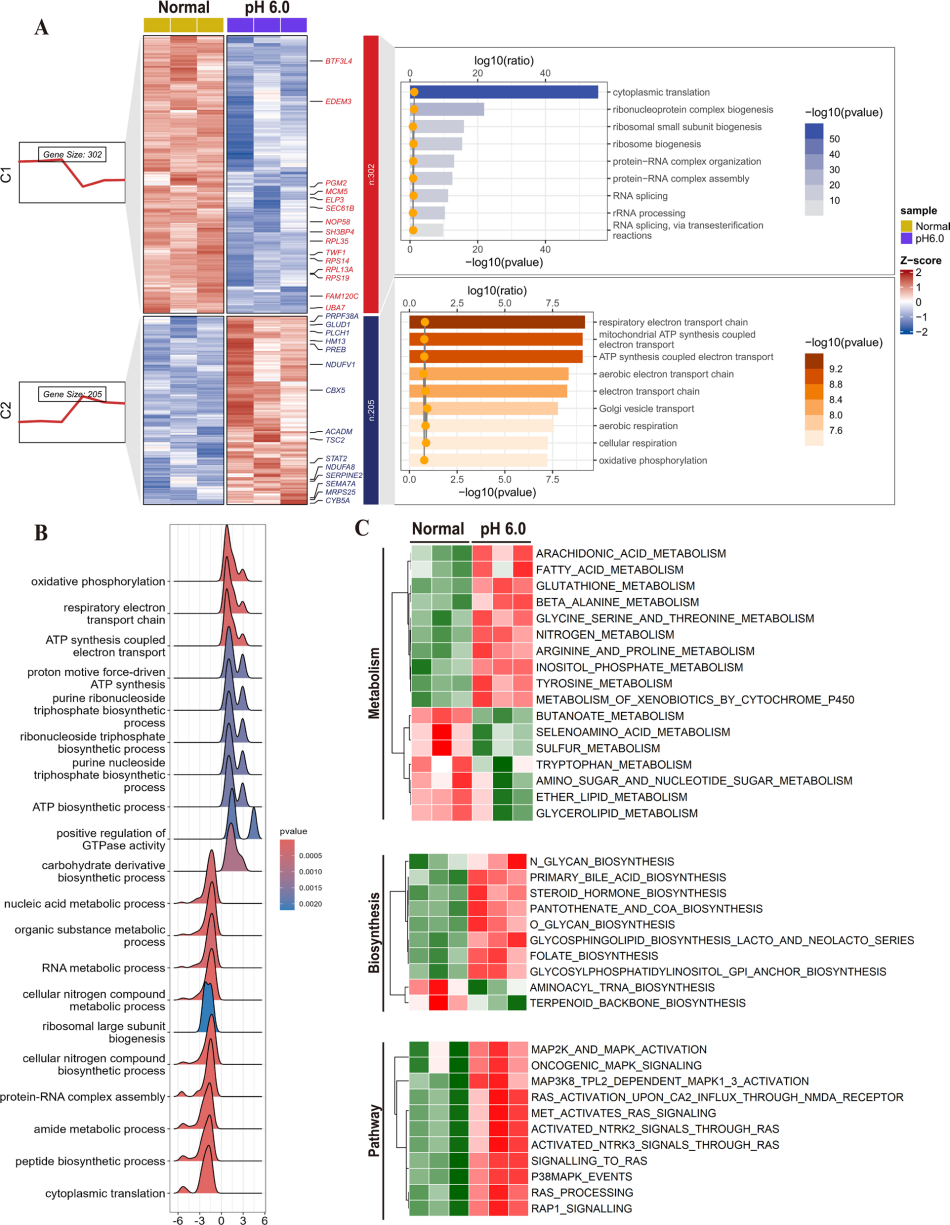
Proteomics characterized the cellular functional status of RASFs after acid stress for 6 h.** A** Differentially expressed proteins identified in proteomics were clustered according to FCM algorithm, and the clusters were enriched based on KEGG database and GO database. **B** Ridge plot presented the status of the GOBP pathways of RASFs which was identified by GSEA. **C** The status of metabolism, biosynthesis, and pathways in RASFs after extracellular acidification stimulation was identified by GSVA based on the REACTOME dataset

Proteomics was employed to ascertain the cell functional status of acid-stimulated RASF at 6 h. The differentially expressed proteins are presented through a volcano plot (Supplementary Fig. 4 A). Based on the FCM algorithm, the differentially expressed proteins were classified into two distinct clusters. GOBP enrichment showed that the cluster with an upregulation trend was significantly enriched in Golgi transport, aerobic respiration, ribonucleotide metabolism and synthesis, while the cluster with a downregulation characteristic was significantly enriched in cytoplasmic translation and ribosome formation and assembly (Fig. 6A). KEGG enrichment analysis based on all differential proteins unveiled oxidative phosphorylation, ribosome function, protein transport, and alterations in various metabolic pathways of RASF (Supplementary Fig. 4B). The screening of GO Biological Process (GOBP) and GO Cellular Component (GOCC) demonstrated significant activation of bioprocesses associated with energy synthesis, encompassing oxidative phosphorylation and ATP production, while indicating significant inhibition of ribosome synthesis and assembly as well as nucleotide metabolism (Fig. 6B). The cellular components of mitochondria and endoplasmic reticulum apparatus were significantly upregulated, while ribosomes and nucleotides were significantly downregulated (Supplementary Fig. 4 C). Notably, acidification significantly negatively regulated intracellular non-membrane-bounded organelle. The constructed enrichment pathway network shows that short-term acidification stress mainly affects RNA metabolism and the part of cell energy metabolism related to mitochondria (Supplementary Fig. 4E). GSEA enrichment based on the KEGG database indicated the activation of oxidative phosphorylation and reactive oxygen generation, along with RNA retention and the inhibition of the ribosomal pathway, while activation of RAP1 and cell adhesion molecular pathways was also observed by GSEA (Supplementary Fig. 4D). The REACTOME dataset was utilized for GSVA analysis, and the functional alterations of RASF were delineated in relation to metabolism, biosynthesis, and signaling pathways (Fig. 6C). The results revealed that the metabolism of fatty acids and specific amino acids was markedly activated, the biosynthesis of glycolipids and glycosides was considerably enhanced, and the activation of the signaling pathway represented by RAS-MAPK was observed. Ultimately, the network maps of oxidative phosphorylation and RAS-MAPK pathways in KEGG enrichment were fabricated, and the outcomes demonstrated that these pathways were interrelated (Supplementary Fig. 4 F).

### Acid stimulation activates the AP1-centered transcription factor network in RASF

**Fig. 7:**
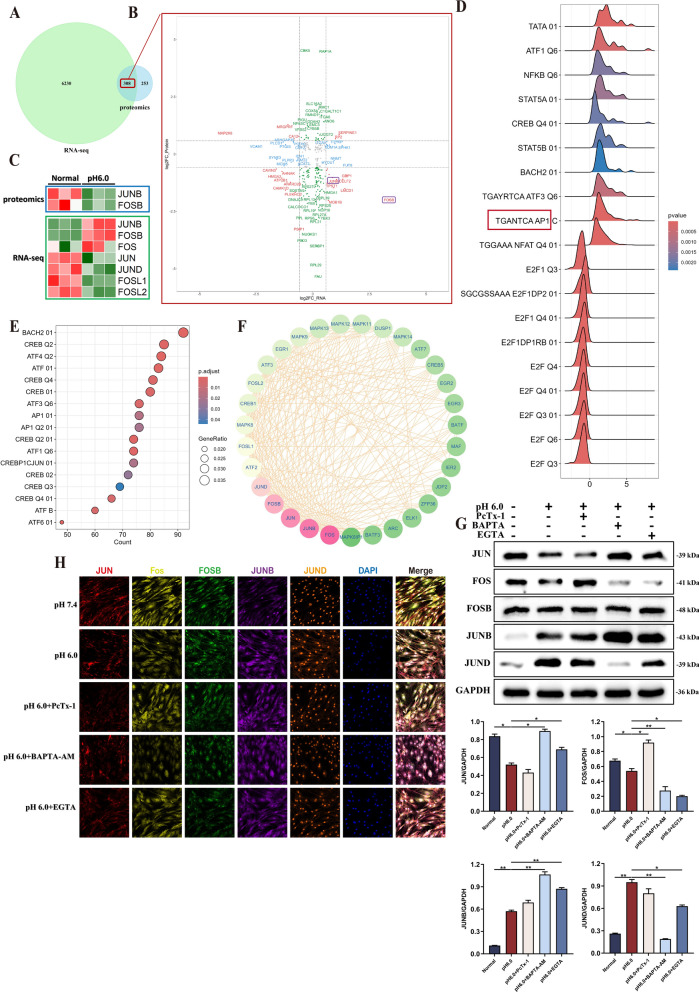
Transcription Factor Network in RASFs after extracellular acid stimulation. **A** The Venn diagram illustrated the intersection of DEGs between the proteomics and RNA-seq data. **B** The association between transcriptional and translational processes was analyzed across nine quadrants. **C** Heatmap presented the subunits of transcription factor AP1 in proteomic and RNA-seq. **D** Ridge plots displayed the transcription factor target enrichment analysis of RNA-seq by GSEA.** E** Enrichment of transcription factor targets for upregulated genes in RNA-seq. **F** Central transcriptional status of the transcription factor AP was identified by network analysis.** G** The expression of AP1 subunits in RASF was confirmed by Western blotting in the presence of 100 nM PcTx- 1, 1 mM EGTA and 10 μM BAPTA-AM. **H** The expression and localization of the AP1 subunit in RASF treated with 100 nM PcTx- 1, 1 mM EGTA, and 10 μM BAPTA-AM were confirmed by multiple immunofluorescence (n = 3). Scale bar = 50 μm, magnification = 200x. Data are presented as the mean ± SD from three independent experiments. Statistical significance was determined by one-way ANOVA followed by Dunnett’s multiple comparison test: *P < 0.05, **P < 0.01, ns: not significant

The intersection of transcriptome and proteome DEGs was conducted (Fig. 7A). To explore the temporal variations of gene expression, the intersection was distributed into nine quadrants (Fig. 7B). The compositional alterations of FOSB and JUNB genes were observed in the ninth quadrant. Both FOSB and JUNB are subunits of the transcription factor AP1, which has five subunits: JUN, JUNB, JUND, FOS, and FOSB (Fig. 7C). These subunits are constitutively activated by binding to various transcription factors like NFκB, ATF and NFAT to maintain transcriptional stability. GSEA enrichment based on the PID dataset revealed significant activation of pathways like AP1 PATHWAY, NFAT PATHWAY, MAPK TRK PATHWAY, and TCR RAS PATHWAY in the proteomics (Supplementary Fig. 5 A). GSEA enrichment was performed on transcriptome data based on transcription factor target (TFT) dataset, and ridge plots were constructed to select positive and negative enriched transcription factors. Significant activation was observed in transcription factors including AP1, NFκB, ATF, and NFAT (Fig. 7D). Analysis of the enrichment of up-regulated genes in the transcriptome revealed that a significant number of up-Regulated genes were regulated by the transcription factor AP1 and the related transcription factors CREB and ATF, based on the TFT dataset (Fig. 7E). Ultimately, the GSE89408 dataset was enriched with reference to the PID database, and the AP1 PATHWAY, NFAT PATHWAY, STAT4 PATHWAY, and ATF2 PATHWAY were prominently activated (Supplementary Fig. 5B). The interaction between AP1 and numerous subunits of transcription factors, such as MAPK, CREB, and ATF, was affirmed through the construction of transcription factor networks (Fig. 7F). Furthermore, the AP1 subunit was quantitatively determined through western blotting (Fig. 7G). The findings indicated that the total FOSB protein remained stable following acid stimulation and calcium chelation. The expression of JUN and FOS declined under acid stimulation, while the expression pattern was completely reversed after the treatment with PcTx- 1 and the calcium ion chelator. The expression of JUNB and JUND increased after acid stimulation, and the expression of JUNB was further enhanced by inhibitors and chelators, while the expression of JUND was suppressed. Ultimately, immunofluorescence staining demonstrated significant variations in the expression of AP1 subunits subsequent to acid stimulation, yet only the JUND and FOSB subunits were conspicuously enriched within the nucleus (Fig. 7H).

## Discussion

**Fig. 8:**
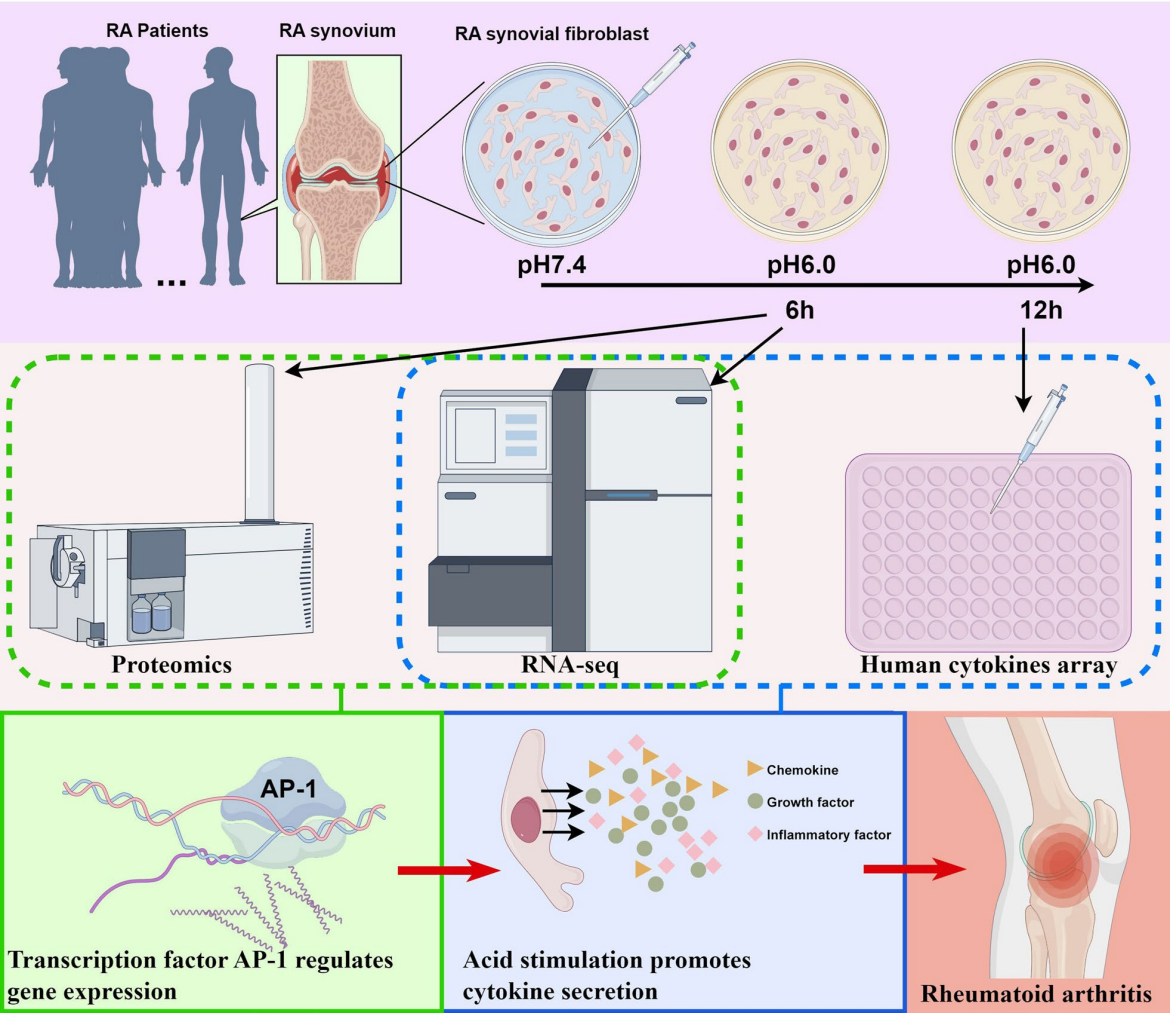
Acidosis promotes cytokine secretion and induces synovial inflammation through transcription factor AP1

This study aims to examine the impact of acidosis on the immune microenvironment of RA by analyzing cytokine secretion and the expression of co-stimulatory factors in RASF. The current study has yielded several key findings. Firstly, cytokine secretion and T cell activation in RASF were significantly enhanced following acid stimulation, with lymphocyte infiltration being promoted through increased chemokine secretion and angiogenesis. Secondly, the expression of inhibitory co-stimulatory molecules and antigen-presenting molecules was significantly elevated after acid stimulation, facilitating the formation of a complex immune landscape. Thirdly, mitochondrial function in RASF demonstrated significant enhancement after short-term acid stress, accompanied by a reconfiguration of metabolic patterns. Fourthly, the transcription factor AP1 was significantly activated following acid stimulation and regulated RASF gene expression through the transcription factor network (Fig. 8).

Firstly, the cytokine secretion and T cell activation of RASF were significantly enhanced following acid stimulation, and lymphocyte infiltration was promoted through the enhancement of chemokine secretion and angiogenesis. This study supports a novel mechanism in which the homeostatic alterations of extracellular pH regulate the release of cytokines and induce the activation of early immune responses. Acidosis-induced RASF elevates the transcription levels of numerous cytokines, encompassing classical innate immune cell chemotactic combinations such as CCL2, IL8, and CCL5, along with inflammatory cytokine combinations like IL6 and IL12, which are contingent upon adaptive immune responses (Casanova et al. 2024). Analysis of the differential expression of supernatant cytokines following acidizing stimulation revealed that acidizing stimulation significantly enhanced the chemotaxis of immune cells, which might be the crucial factor for the accumulation of inflammatory cells in the joint cavity during the early stage of RA (Baker et al. 2021). In addition, a variety of vascular growth factors and fibroblast growth factors showed stronger signals in the control group and were further elevated after acid stimulation (Lee et al. 2023). Abundant neovascularization not only furnishes the foundation for the impairment of the disease structure of RA, but also facilitates the infiltration of inflammatory cells into the joint. Certainly, we have also observed that the qRT-PCR results corresponding to the HCA time were inconsistent with the HCA, particularly manifested conspicuously in genes such as FGF7, ANGPT- 1, CXCL12, and IL- 17 A. Apart from the potential molecular mechanisms we previously mentioned, the inhibitory feedback of autocrine signaling could also be one of the causes for this (Nguyen et al. 2017). After 12 h of accumulation, the extracellular cytokine concentration should be regarded as a significant factor in regulating the inflammatory phenotype of synovial fibroblasts. The overexpression of cytokine genes like IL6 and VEGF, as well as the ineffectiveness of PcTx- 1 and calcium chelators, might also be attributed to the influence of autocrine signaling (Nguyen et al. 2017). Under such circumstances, the extracellular acidic signal played a role in triggering the cyclic secretion under inflammation. Conclusively, acidification stimulation primarily augmented the expression of chemokines rather than inflammatory factors and conspicuously facilitated angiogenesis and tissue growth. These results indicate that acidosis could be a significant factor in the early immune cell invasion of RA and the pathological deterioration of joints subsequent to drug treatment.

Secondly, the expression of inhibitory co-stimulatory molecules and antigen-presenting molecules was significantly augmented after acid stimulation to facilitate the formation of a complex immune landscape. Several research have shown that extracellular acidification directly impairs the anti-tumor immune response and T cell function is restored after pH neutralization treatment (Nakagawa et al. 2015). Immune checkpoint therapy has revealed that tissue acidification reduces the effectiveness of multiple targeted drugs (Knopf, et al. 2023; Huntington, et al. 2022). We observed that extracellular acidification significantly upregulates the expression of ICAM1 and CD274 (PDL1), while concurrently downregulating the expression of CD28, ALCAM, and other genes. This suggests that extracellular acidification may influence the phenotype of CD8^+^ T cells in the synovium by modulating the expression of co-stimulatory molecules. As previously discussed, the GzmK^+^ CD8^+^ T cell subset in the synovium constitutes a distinct population of T cells marked by elevated expression levels of genes associated with senescence and exhaustion, as well as those involved in the secretion of pro-inflammatory cytokines. (Jonsson, et al. 2022; Zhang et al. 2019; Mogilenko et al. 2021). We postulate that in the milieu of acid-stimulated cytokine-rich microenvironment and the high expression of PDL1, GzmB^+^ T cells are more inclined to undergo apoptosis induced by PDL1 after TCR binding, which leads to a reduction in the distribution of cytotoxic T cells in synovium. Meanwhile, the GzmK^+^ CD8^+^ T cell subsets is less impacted. The GzmK^+^ CD8^+^ T cell subtype is less susceptible, and may even be the result of differentiation after T cells receive PDL1 signals under the influence of pro-inflammatory cytokines, thereby being enriched in synovium. Additional evidence indicates that GzmK^+^ T cells are not only abundant in various autoimmune diseases but have also been presented as a typical functionally exhausted CD8^+^ T cell phenotype in single-cell data studies of pan-cancer (Zheng, et al. 2021). Although some studies have proposed that significant improvements were observed in the CIA models treated with the strategy of overexpressing PDL1, the PDL1-PD1 signaling axis has recently been discovered to facilitate the humoral immune response by maintaining the survival and activation of B cells, which is the most intractable problem in RA (Ogishi et al. 2024; Wood et al. 2024; Wang, et al. 2023). Here, we posit that acid stimulation in RA alters the direction of the entire immune landscape in synovial tissue via the secretion of proinflammatory cytokines and the expression of inhibitory co-stimulatory molecules.

Thirdly, mitochondrial function demonstrated significant enhancement in RASF after short-term acid stress, accompanied by the reconfiguration of metabolic patterns. Our proteomic data provide support for the direct effect of extracellular H^+^ inflow on mitochondrial function. (Zhang et al. 2020). Primarily, the expression of COX5 A and COX7 A2 proteins significantly increased. COX5 A and COX7 A2 are constituent subunits of complex IV, which transfer electrons to oxygen and consume hydrogen ions to form water. During this process, the free hydrogen ions are immobilized. (Vercellino and Sazanov 2022; Decker and Funai 2024). Furthermore, the expression of growth hormone-induced transmembrane protein (GHITM) in the inner mitochondrial membrane was markedly elevated. GHITM mediates proton-dependent mitochondrial calcium efflux by transferring calcium ions from the mitochondrial matrix and H^+^ into the mitochondrial matrix (Patron et al. 2022). The H^+^ in the mitochondrial matrix can function as a substrate for complex 4, providing a proton gradient and reducing mitochondrial damage via the reaction with reactive oxygen species (Vercellino and Sazanov 2022). Interestingly, our previous research showed that acid stimulation caused mitochondrial Ca^2+^ overload and induced mitochondrial damage in chondrocytes (Zai et al. 2022). These data indicate that mitochondria might be the crucial organelles for maintaining intracellular H + homeostasis under acid stimulation. (Hernansanz-Agustin et al. 2024). On the other hand, ribosomes exhibit a reduction in both protein subunits and ribonucleotides. Most crucially, the component enrichment of membraneless organelles exhibited marked downregulation, indicating that intracellular Ion homeostasis directly impact the function of these organelles. Furthermore, we observed a decline in the metabolism of ribonucleotides and RNA, while the biosynthesis of ribonucleoside triphosphates was augmented. This indicates that free RNA might participate in the energy metabolism pathways dominated by mitochondria via phosphorylation (Hibbs et al. 2016). Meanwhile, the augmentation of amino acid and lipid metabolism pathways may serve as a compensatory mechanism for energy metabolism under acid stress (Kandasamy et al. 2018; Liu et al. 2025). Several studies have demonstrated that extracellular low-concentration lactic acid significantly enhances the mitochondrial metabolic capacity of astrocytes via ASIC1a and reduces the production of ROS by promoting lactic acid metabolism (King, et al. 2021). It is notable that the transcriptome and proteome did not register data on carbonic anhydrase (CA), the most critical component of the systemic acidosis response, which is associated with the pH buffering employed in the DMEM medium (Sheth 2004).

Fourthly, the transcription factor AP1 was significantly activated after acid stimulation and regulated RASF gene expression through the transcription factor network. This result is not unexpected since it is widely recognized that AP1 signaling is markedly regulated either directly or indirectly by intracellular Ca^2+^ (Santos et al. 2006; Yeh and Parekh 2018). We also ascertained that within 6 h of acidification stimulation, the outcomes resulting from the ASIC1a inhibitor PcTx- 1 and the calcium chelator were disparate. This could potentially be ascribed to the perturbation of H + signals, the regulation of intracellular calcium pools, and the functions of other proton exchangers. It is notable that the expression of JUNB was significantly elevated with the application of calcium ion chelators, suggesting that JUNB might be predominantly regulated by the H^+^ mediated signal, while the Ca^2+^ signal plays an opposite role in this process, the signal crosstalk between these two ions has been witnessed for the first time. spatial transcriptomics studies have indicated that the lining RASF, comprising the active lining RASF, the resting lining RASF, and the activating/resting interphase lining RASF, have all exhibited enrichment levels of AP1 subunits such as FOS, JUN, JUND, and JUNB in the accessible chromatin region during ATAC analysis (Smith et al. 2023). The contributions of transcription factors such as AP1, CREB5, and ATF7 to the regulation of downstream genes of growth factors and immune receptor signals (such as IL- 1R1) were observed. (Smith et al. 2023). This supports our study that the activation of acid-induced transcription factor AP1 leads to immune cell enrichment in RA by regulating cytokine secretion. However, we also observed discrepancies between the proteomics data and the results derived from immunological experiments. This may be attributed to technical limitations, specifically the insufficient resolution of label-free proteomics in distinguishing low-molecular-weight protein subunits (Jin et al. 2008). Due to the lack of a separate reference library, the features required for precise annotation were inadequate, leading to the merging of molecules from the same family. Therefore, we deliberately opted for re-quantification.

One limitation of this study is that although we emphasize that acidification stimulation can promote the secretion of cytokines in RASF, changes in epigenetic status are generally considered the main cause of the inflammatory phenotype in rheumatoid arthritis synovial tissue (Ospelt 2023; Buch et al. 2021; Ohkura and Sakaguchi 2020). However, there is currently insufficient data on whether acidification stimulation is involved in the epigenetic regulation of RA synovial tissue. Some studies have shown that acidification stimulation can regulate post-translational modifications of proteins, such as acetylation Corbet et al. 2016. We were inspired by the GSVA analysis based on REACTOME, which indicated that acidification stimulation leads to a decrease in the levels of histone H3k4 and histone arginine methylation. However, whether these are related to epigenetic alterations associated with the disease pathology of RASF remains to be further investigated. Notably, in a single-cell study of synovial tissue on the 9 th day of mouse serum transfer arthritis, a unique fibroblast subtype was discovered, with overexpressed genes related to “acid secretion” and “hydrogen ion transport” (Croft et al. 2019). Interestingly, the GO analysis of this subpopulation indicated significant enrichment in cellular functions such as inflammatory response, MHC class I antigen presentation, calcium ion signaling, and mitochondrial aerobic respiration complexes (Croft et al. 2019). However, in the same study, the cytokine secretion phenotype was significantly enriched in other subpopulations. Although studies on this subpopulation may reveal the impact of the extracellular acidic environment on the epigenetics of RASF, unfortunately, there are currently no high-resolution and high-throughput data based on human samples reporting on this subtype. These studies usually focus more on the distinction between the lining and sub-lining layers of the synovium (Zhang et al. 2023; Collins et al. 2023; Wei, et al. 2020). On the other hand, the disease context of RASF itself may be an important feature of this study. We demonstrated the significant potential of extracellular acidification as an inducer of inflammatory activation and amplification of inflammatory signals in RASF by simulating the in vivo acidic environment. These potentials are manifested not only in the activation of intracellular pathways and transcription factors of RASF by the extracellular acidification environment through surface receptors such as ASIC1a, but also in that the cytokines secreted by RASF upon acidification stimulation may self-activate through autocrine signalling, particularly when the accumulation of these cytokines leads to an increase in concentration, and extracellular acidification stimulation may accelerate this process. However, the interaction between the extracellular acidic environment and the immune environment in the actual pathological state still needs to be further explored in vivo, including the use of drugs such as sodium bicarbonate to regulate pH and evaluate the disease status. This is also a key limitation of this study.

In summary, this research evaluated the regulatory impacts of acid stimulation on the extracellular cytokine secretion and intracellular signal response of RASF by means of a multi-omics approach, and verified that acidosis facilitates the development of autoimmune diseases dominated by RASF. However, the role of acidosis in RA still requires comprehensive consideration. Studies have also shown that acidosis inhibits T cell activation, but significantly promotes the activation of innate immune cells and antigen presentation (Cheng et al. 2023; Coutant 2021). Therefore, understanding the cell markers stimulated by acidosis and the spatial distribution of synovial tissue may be the key to decryption. We anticipate that this study could furnish more detailed information for the research on acidosis in autoimmune diseases and offer more concepts for the clinical treatment of RA.

## Supplementary Information

Supplementary material 1: Supplementary Figure. 1. Functional enrichment of bulk-RNAseq in synovial tissues of rheumatoid arthritis. **A.** Multiple volcano plots displayed differential expression gene identified in the GSE89408 datasetaccording to disease staging. **B**-**D**. The status of cytokine, immuneand T cell regulationpathways in RA synovium was identified by GSEA

Supplementary material 2: Supplementary Figure. 2. Functional enrichment analysis of RNA-seq data for RASF following acidification stimulation at pH 6.0. **A.** The volcano plot displayed the differentially expressed genes identified in RNA-seq. **B. **KEGG pathway enrichment analysis of DEGs in RNA-seq data. **C**-**D**. The status of the cytokineand T cell regulatorypathways of RNA-seq was identified by GSEA. **E.** The status of the immune activation and T cell activation pathways in RASFs was identified by GSVA

Supplementary material 3: Supplementary Figure. 3. Functional enrichment analysis of HCA data for RASF following acidification stimulation at pH 6.0. **A. **Quantification of the expression in the negative control and positive control of HCA. **B.** GO enrichment analysis of the top 60 ranked cytokines in normal group. **C.** GO enrichment analysis of differential cytokine expression. **D. **The activation of cell chemotactic pathways by differential cytokine expression was identified by GSEA. **E.** Distribution of HCA plates. The data shown are the mean ± SEM. The data were analyzed by One-way ANOVA and Dunnett multiverb tests, “ns” means no significant difference

Supplementary material 4: Supplementary Figure. 4. Functional enrichment analysis of Proteomics data for RASF following acidification stimulation at pH 6.0. **A.**The volcano plot displayed the differentially expressed proteins identified in proteomics. **B.** KEGG pathway enrichment analysis of differential expression proteins in RASFs. **C.** Ridgeline plots presented the status of the GOCC pathways of RASF which was identified by GSEA. **D.** KEGG pathway enrichment analysis of differential expression proteins in RASF was identified by GSEA. **E.** The association network between RNA metabolism and mitochondrial-related energy metabolism. **F.** The association network between oxidative phosphorylation and several activated signaling pathways

Supplementary material 5: Supplementary Figure. 5. Status of AP1 and related pathways. **A.** Enrichment analysis of AP1 and related pathways in differentially expressed proteins from the Proteomics was performed by GSEA based on the PID dataset. **B.** Enrichment analysis of AP1 and related pathways in DEGs from the GSE89408 dataset was performed by GSEA and the PID dataset

## Data Availability

No datasets were generated or analysed during the current study.

## Abbreviations

RA: Rheumatoid arthritis
RASF: Rheumatoid arthritis synovial fibroblast
ASIC1a: Acid-sensing ion channels 1a
FCM: Fuzzy C-Means
PcTx- 1: Psalmotoxin 1
EGTA: Ethylenebis (oxyethylenenitrilo)tetraacet
MTX: Methotrexate
DMARDs: Disease-Modifying Antirheumatic Drugs
KEGG: Kyoto Encyclopedia of Genes and Genomes
GO: Gene Ontology
VEGF: Vascular endothelial growth factor
NFAT4: Nuclear factor of activated T cells
AP- 1: Activator protein- 1
GSEA: Gene Set EnrichmentAnalysis
GSVA: Gene set variation analysis
